# 

**Published:** 1889-07-20

**Authors:** 


### The Student's Column.

### MEDICAL JURISPRUDENCE FOR INDIA.

The author states that in compiling this book* his object has been to supply a want often felt while lecturing on medical jurisprudence. That such a want existed, especially in India, there can be no manner of doubt, for nothing of the kind has been produced since the late Dr. Chevers published on the same subject some quarter of a century ago. Brigade-Surgeon Lyon, after his many years' experience as chemical analyser to Government, has admirably supplied this want. He has also supplemented his own large experience by drawing on medico-legal reports made by other Indian officers, the result being a concise and complete manual on the subject, which while specially adapted for use in India contains all the information on the subjects treated of required in any other country. There is very little difference between the medical element of medical jurisprudence in any country, but inasmuch as the law of India and the law of England are not the same as regards many subjects, some special information is required for the former country. That the information given in Dr. Lyon's work is correct, Mr. Inverarity's name is amply sufficient voucher.

The work extends to some five hundred and fifty pages, and therefore it is impossible to review it thoroughly in the columns of a hebdomadal journal. The first chapter deals with the law of evidence, the second with general questions connected with the dead body, and the third with wounds and mechanical injuries. Then we have drowning, hanging, suffocation, and strangulation, burns, and spontaneous combustion. Here we pause, and turn to see what the author has to say on the *questio vexata* of spontaneous combustion. It will perhaps be recollected that when, years back, Charles Dickens in one of his works introduced a case of spontaneous combustion the possibility of such an occurrence was denied, whereupon Dickens cited several apparently authentic cases. So, until the present time, the matter has remained *sub judice*. Dr. Lyons's opinion is as follows: "A consideration of the circumstances recorded in cases of this class fails to support the hypothesis of spontaneous combustion." Death from heat, cold, electricity, and starvation are next treated of. In connection with this latter subject tables are given showing the nutritive value of articles of food used in India. We think an addition to this should be made in future editions, so that what have been called the famine foods of India might also be exhibited—meaning the grass seeds, barks, and roots to which the people resort when grain is unobtainable. All kinds of poisons are treated of at great length, chapter xxv. being devoted to snake poisons and ptomaines. It is known that the mortality in India is considerable from snakebite, but it is also questioned if snakes are responsible for all the deaths attributed to them. The author relates a case bearing on this point: "The body of a man was sent for examination, accompanied by the body of a snake, which, it was alleged, had bitten him. On examination the snake proved to be a non-poisonous one, and the cause of death strangulation." There is no doubt that in India deaths are often reported as from snakebite, either because the true cause is unknown, or with the view of concealing how death occurred. It is mentioned by the author that in India criminals were sometimes executed by snakebite. Only one case of homicide by snakebite has been recorded in recent times. We cannot follow the author through his chapters on "Unnatural Offences," "Inheritance and Legitimacy," "Infanticide Life Assurance," "Insanity," etc. Neither can we do more than refer to the copious appendix in which, among other subjects, is given full instruction as to *post mortem* examination. The work is illustrated by excellent plates, the type is clear, and the "get up" of the volume strong, as required in a tropical climate. It is not too much to say that Dr. Lyon's medical jurisprudence is a necessity for every medical man, and for every lawyer practising in the tropics, and will be found almost equally useful in any part of the world.

* "A Textbook of Medical Jurisprudence for India." By I. B. Lyon, F.C.S., F.I.C., Brigade-Surgeon Bombay Medical Service, Chemical Analyser to Government, Professor of Chemistry and Medical Jurisprudence, Grant Medical College, Bombay, Fellow of the University of Bombay. Revised as to legal matters by J. D. Inverarity, Esq., of the Inner Temple, Barrister-at-Law, and Advocate of the High Court, Bombay. W. Thacker and Co., 87, Newgate-street.

### UNIVERSITY OF LONDON, M.B. PASS EXAMINATION, MAY, 1889.

First Division: Robert James Carter, King's College; Edward Augustus Roberts, St. Thomas's Hospital; Henry Sharman, University College; John King Warry, London Hospital.

Second Division: J. Oglethorpe W. Barratt, *B.Sc.*, University College; Samuel Bird Cook, St. Thomas's Hospital; Cecil Whitehall Cooke, St. Thomas's Hospital; Charles Seymour Dowdell, University College; Nathan Charles Haring, Owens College and Manchester Royal Infirmary; William Henry Iddon, Owens College; William Joberns, Queen's Birmingham and London Hospital; Cecil Price-Jones, Guy's Hospital; William Harry Kelson, London Hospital; William Henry Tomlinson, Owens College.

### SPECIAL ANNOUNCEMENT.

### MISS FLORENCE NIGHTINGALE.

The next (August) monthly part of The Hospital, and the weekly issue for 27th July, will contain a biographical sketch of Miss Florence Nightingale, tracing the remarkable career of that devoted lady, and showing the effect of her labours at the present time. The article in the August number will be accompanied by a portrait of Miss Nightingale, which has been reproduced at great cost and perfection; and it will at last be possible for every member of the nursing profession to possess the likeness of one who, by a life of self-sacrifice and philanthropic labour, has raised herself to pre-eminence among women. The monthly number will be issued at sixpence as usual, but orders should be sent early.

### NOTICE TO CORRESPONDENTS.

All MS., Letters, Books for Review, and other matters intended for the Editor, should be addressed The Editor, The Lodge, Porchester Square, London, W.

### Notice to Advertisers and Subscribers.

All Advertisements, Orders for Copies of The Hospital, and Business Communications should be addressed to The Publisher, *not to the Editor*, The Hospital, 139-140, Salisbury-court, Fleet-street, E.C.

Vol. V., now ready, 4s., post free. Cases for binding, may be had of the Publisher, at 1s. 6d. each.

### To Residents, Secretaries, and Matrons.

The Editor will be glad to have the Regulations and each Report as issued by Asylums, Hospitals, Convalescent and Nursing Institutions, as well as those of other Charities, and an early copy in duplicate of all Appeals and Festival Notices, etc., addressed to *The Editor, The Lodge, Porchester-square, London, W.* Any superintendent, secretary, matron, or other chief official who regularly sends such information may be registered, on application to the Publisher, to receive free of cost a cover for binding The Hospital in half-yearly volumes.

### Amusements and Relaxation.

### Second Quarterly Word Competition Commenced July 6th, ends September 28th.

Three Prizes of 15s., 10s., 5s., will be given for the largest number of words derived from the words set for dissection.

Proper names, abbreviations, foreign words, words of less than four letters, and repetitions are barred; plurals, and past and present participles of verbs, are allowed. Nuttall's *Standard* dictionary only to be used.

N.B.—Word dissections must be sent in WEEKLY, arranged alphabetically, with correct total affixed.

The word for dissection for this, the *Third* week of the quarter, being

"BRIGHTON."

**Results of First Week.**

| Names. | July 11th. | Totals. | Names. | July 11th. | Totals. |
|---|---|---|---|---|---|
| Nurse Duty | 31 | 31 | Isabel | 26 | 26 |
| N'importe Qui | 30 | 30 | Buxton | 25 | 25 |
| Jumps | 30 | 30 | Nux Vom | 25 | 25 |
| Tinie | 30 | 30 | Qu'appelle | 25 | 25 |
| Patience | 30 | 30 | Spes | 24 | 24 |
| Amicus | 30 | 30 | Secnarf | 23 | 23 |
| Lauriston | 29 | 29 | Leirion | 22 | 22 |
| Broxbourne | 29 | 29 | Matron | 21 | 21 |
| Lightowlers | 29 | 29 | F. C. J. | 19 | 19 |
| Speedwell | 28 | 28 | Percy Verance | 18 | 18 |
| Rattler | 28 | 28 | Gleniffer | 18 | 18 |
| Sister | 28 | 28 | Gypse | 17 | 17 |
| Paignton | 27 | 27 | Ladyr | 17 | 17 |
| Fassifern | 27 | 27 | Nurse Amie | 15 | 15 |
| W. R. E. | 27 | 27 | A. B. | 15 | 15 |
| Esperance | 26 | 26 | W. G. R. | 15 | 15 |
| M. W. | 26 | 26 | Essie | 10 | 10 |

**Notice to all Correspondents.**

N.B.—All letters referring to this page which do not arrive at 139 and 140, Salisbury-court, London, E.C., by the *first post on Thursdays*, and are not addressed PRIZE EDITOR, will in future be disqualified and disregarded

### Scraps and Gleanings.

Hospital Sunday at Milford-on-Sea resulted in a collection of over £12.

Coventry has now 45 working men as governors on its Hospital Board.

At St. Saviour's Church, Forest Hill, £19 was collected on Hospital Sunday.

Watlington Cottage Hospital received £51 last year in patients payments.

Dr. P. S. Abraham has an article in the *Fortnightly Review* on "Leprosy."

Dr. Prosser James has resigned his lectureship at the London Hospital Medical School.

Dr. Boxall has been appointed assistant obstetric physician at the Middlesex Hospital.

The Congregational Church, Finchley, maintains a cot at the East London Hospital for Children.

Councillor Furness made a good speech on Hospital Sunday at West Hartlepool. £100 was collected.

The Goldsmiths' Company have undertaken the whole burden of starting the Polytechnic at New Cross.

Edinburgh Royal College of Physicians has given £250 to the Victoria Nurses' Jubilee Institute.

The Sheriff of Hull (Mr. Wilson) has offered £1,000 towards the proposed Victoria Children's Hospital.

A concert was given at the National Hospital (Albany Memorial) on Thursday in aid of the Chapel Fund.

The Central London Ophthalmic Hospital had a concert given in its aid at Steinway Hall on Wednesday.

Some doubt is now expressed as to whether Yoxall really is a leper, or is only suffering from symmetrical gangrene.

The Lady Superintendent of Guernsey Convalescent Home appeals for another £100 a year, offering to subscribe £20 herself.

The Princess Louise had a good reception at Maidstone when she opened the new wing of the West Kent General Infirmary.

The annual report of Royston College Hospital tells of 52 patients treated last year at an average cost per day per patient of 2s. 9d.

An appeal for funds is issued by the Rev. G. W. Lawson, who is mission clergyman in the new parish of St. Thomas, Kensal Town.

At the Moravian Chapel, Fetter-lane, £5 was collected on Hospital Sunday. The Rev. J. A. Porter preached on "The Cry for Mercy."

The programme of the proceedings of the British Medical Association at Leeds in August, appeared in last week's *British Medical Journal*.

The Al Fresco Fayre is to be reproduced at Margate in August, on behalf of the Convalescent Home of the Victoria Hospital for Children.

M. Pasteur has written to the Lord Mayor, acknowledging the receipt of the resolution passed at the recent Mansion House meeting.

The Foresters' Hospital Sunday at Cheltenham was kept on July 6th. The Rev. C. V. Childe preached, and the collection nearly reached £12.

The Evelina Hospital has a whooping-cough ward. This cannot be too widely known, for hundreds of children die annually from whooping-cough.

A grocer at Lincoln has withdrawn his subscription to the County Hospital, because the samples of groceries he sent in were never even opened.

Two hospital cases are exciting interest in the provinces; that against Dr. Conry, of Hope Infirmary, and that against the hospital authorities at Elgin.

Dr. Julia Mitchell is attending the French Congress. It is a pity someone with fewer crotchets could not have been found to represent England.

The Lord Mayor presided over a large meeting at the Mansion House on July 9th, in aid of the National Association for Supplying Female Medical Aid to India.

The Princess Mary Village Homes were visited last week by a large number of friends and supporters. A satisfactory report was read by Mrs. Meredith.

On July 10th Northfields Infectious Diseases Hospital was opened by Lord Edmond Fitzmaurice. It cost £2,000, the site having been given by Lord Lansdowne.

The Infectious Diseases Hospital at Grantham was destroyed by fire last week. The building was a wooden structure and the property of the Grantham Corporation.

St. Peter's, Greenwich, collected £5 14s. on Hospital Sunday. The Rev. F. Storer Clark preached in the evening on the Samaritan's compassion given as a pattern.

The Worshipful Company of Goldsmiths have voted £100 to the Children's Country Holidays Fund (office, 10, Buckingham-street, Strand), of which the Hon. Alfred Lyttelton is treasurer.

Clissold Park, in the north-eastern suburbs, is to be opened to the public on the 24th inst., the opening ceremony being performed by Lord Rosebery, as chairman of the London County Council.

An Order in Council appears in the *Gazette* in respect to rabies and the muzzling of dogs in the City and Metropolitan Police districts. The order is to take effect from the 31st inst., and to remain in force till the end of the year.

Prince George of Wales presided at the inaugural banquet of the Sea Fishermen's Provident Fund, held by permission of the Fishmongers' Company at Fishmongers' Hall. The toast of "The Queen," the patroness of the fund, was loyally honoured.

A crowded drawing-room meeting on behalf of the Cyprus Society was held last week at 83, Lancaster-gate, the residence of the Earl and Countess of Meath. Earl Nelson presided. The society has been established for the building of a memorial hospital to General Gordon in Cyprus, and for promoting education in the island.

### The Nursing Mirror.

No. 147.—Vol. VI. SATURDAY, JULY 20, 1889. (GRATIS.)

### Vacancies and Employment Wanted.

**SCALE OF CHARGES FOR ADVERTISEMENTS.**

FOR VACANCIES AND EMPLOYMENT.

| | s. | d. |
|---|---|---|
| Th ee Lines (twenty-four words) ........................... (*Prepaid*) | 1 | 3 |
| For every Eight Words beyond ........................... | 0 | 6 |

10 per cent. deduction for Three Insertions.

All other Advertisements charged as follows:—

| | £ | s. | d. |
|---|---|---|---|
| A Whole Column ........................... | £2 | 15 | 0 |
| A Page ........................... | 5 | 0 | 0 |

| | | |
|---|---|---|
| For 6 insertions, a deduction of ........ | 10 | per cent. discount. |
| ,, 12 or 13 ,, ,, ,, ........ | 20 | ,, ,, |
| ,, 26 ,, ,, ,, ........ | 25 | ,, ,, |
| ,, 52 ,, ,, ,, ........ | 30 | ,, ,, |

Special terms for occasional change of copy during series.

*NOTICE.—Advertisements should* **not be addressed to the Editor,** *but to the Publisher, 139 and 140, Salisbury-court, Fleet-street, E.C , and must be received, accompanied with Stamps or Postal Orders, not later than Wednesday morning for insertion in the same week's issue.*

### CENTRAL LONDON SCHOOL DISTRICT.

NURSES WANTED.

The MANAGERS desire to appoint for their Ophthalmic School at Hanwell FIVE CHARGE-NURSES, who have a certificate of training or have had experience in hospital work, and some knowledge of ophthalmic cases will be advantageous. Salary £26, rising £1 annually to £30 per annum, with rations, lodging, washing, and uniform. Applications to be made on printed forms, which may be obtained at my office, as under, must be returned to me, accompanied by copies of three recent testimonials, on or before the 18th INST.

GEO. E. EAST,
Clerk to the Managers.

10, Basinghall-street, E.C.,

10th July, 1889. (74)

### ST. WILLIAM'S HOSPITAL FOR INFECTIOUS DISEASES, ROCHESTER.

THE ROCHESTER AND CHATHAM JOINT HOSPITAL BOARD require a trained FEMALE NURSE. Salary £25 per annum, payable quarterly, with board, wages, lodging, washing, and uniform.

Applicants must be under 35 years of age and have had experience in similar institutions.

Apply by letter stating age and previous engagements, and with testimonials, before 27th JULY inst., to H. P. MANN, Clerk.

44, High-street, Chatham,

July 10th, 1889. (73)

REQUIRED, Certificated NURSE (trained in Medical, Surgical and Obstetric Nursing), now, for small hospital in the South. Age not under 26. Private cases occasionally. Salary £20 and indoor uniform. Comfortable home and duties usually light. Apply immediately with photo, references and all particulars. Stamped envelope for reply. SISTER, care of Publisher, THE HOSPITAL. (72)

WANTED, a thoroughly trained Medical and Surgical NURSE, for the Nurses' Institute, Jersey. Salary £25. Apply to the LADY SUPERINTENDENT. (6)

HOLLOND INSTITUTION, Nice (Branch, Mentone), — WANTED, for winter, experienced well-educated NURSES. Apply, at once, DIRECTRESS, All Saints' Rectory, Axminster. Also Monthly and General NURSE (August). (3)

ENGAGEMENT DESIRED as Nurse-Companion. Gentleman or Lady. Mental or otherwise. Hospital trained. Domesticated. Doctor's testimonials. References. G., 1, Stamford-street, Old Trafford, Manchester. (33)

CROYDON GENERAL HOSPITAL.—A fully trained NURSE for the Female Wards, and a WARDMAID are REQUIRED for this Hospital. Apply, with testimonials, to the MATRON. (105)

WANTED, NIGHT NURSE for Darlington Fever Hospital. Salary £20 per annum, with uniform. Apply, with copies of testimonials, to MATRON. (106)

WANTED, a respectable PERSON, about 35 years of age, as District Nurse. Must have had some training and experience in sick nursing. Wages £1 per week, and working dress. Apply, stamped envelope, LADY SUPERINTENDENT, 1, Regent-street, Nottingham. (58)

WANTED, for the British Home for Incurables, a properly qualified and trained NURSE, to act as Head Nurse, and take charge of the Home in the absence of the Matron. Salary £35, with board and washing Apply by letter only, with copies of testimonials, to the SECRETARY, British Home for Incurables, 73, Cheapside, E.C. (75)

MAN and WIFE, without Children, desire RE-ENGAGEMENT to take charge Infectious Diseases Hospital. Six years' experience. Have high-class testimonials. Address W. MORPHET, care of R. Hill, Esq., 22, Finch-road, Handsworth, Birmingham. (76)

QUEEN VICTORIA NURSING INSTITUTION, Wolverhampton.—WANTED, immediately, trained NURSES, Medical, Surgical, and Monthly. Apply LADY SUPERINTENDENT. (77)

NURSES.—TWO Certificated Medical and Surgical NURSES WANTED, not over 30. Address, with testimonials, SUPERINTENDENT, 13a, London-road, St. Leonards-on-Sea. (79)

WANTED, by Trained Nurse, a comfortable HOME in Kensington or neighbourhood, would share apartments with another nurse. M., 18, Hermitage-road, Richmond, Surrey. (84)

HOLIDAY ENGAGEMENT REQUIRED by Lady, Trained Nurse. Disengaged one month from beginning of August. Matron's or nurse's duties, or would go abroad. Address C. H., Nurses' Institute, Canterbury. (86)

WANTED, at Stratford-on-Avon Hospital, a thoroughly-trained Medical and Surgical NURSE. Day and night duty taken alternately. Salary £20 to £25, and indoor uniform. Apply to the MATRON. (87)

SOUTHPORT TRAINED NURSES' HOME AND MASSAGE INSTITUTE. — WANTED (immediately) hospital-trained NURSES. Age from 20 to 30. Apply, MRS. STEPHENSON. (85)

WANTED, thoroughly-trained NURSES, for Private Nursing Institution. Salary commencing £25 to £30. Apply, enclosing copies of testimonials and photograph, to the MATRON, Victoria Home for Nurses, Bournemouth. (81)

BRIGHTON BOROUGH FEVER HOSPITAL.—WANTED, HEAD NURSE, for Typhoid Wards. Must be thoroughly competent and capable of training Probationers. Salary £28 per annum, with uniform, board, &c. Apply, stating age, where trained, and enclosing copies of recent testimonials, to MATRON. (82)

LAUNDRYMAIDS.—WANTED, Head and Under LAUNDRYMAIDS. Wages respectively £25 and £20 per annum, with uniform, board, &c. Apply to MATRON, Borough Fever Hospital, Brighton. (83)

HAMPSHIRE NURSES' INSTITUTE, Southampton. WANTED Certificated, Medical, Surgical, and Monthly NURSES. Apply, with testimonials, to HON. SEC. (96)

NURSE ATTENDANT.—RE-ENGAGEMENT by experienced person to elderly invalid or blind. Good reader and needlewoman. Excellent references. M., Saltgate-street, Beccles, Suffolk. (94)

WANTED a NURSE for the Torquay Sanatorium to attend to cases of Infectious Disease. Wages £40 per annum, with furnished room, fire, and light, but exclusive of board. Candidates, who must have had considerable experience with infectious diseases, can apply for further particulars and send testimonials and application to MEDICAL OFFICER OF HEALTH, Town Hall, Torquay. (100)

